# Asymmetric impact of exchange rate on trade between Vietnam and each of EU-27 countries and the UK: evidence from nonlinear ARDL and the role of vehicle currency

**DOI:** 10.1016/j.heliyon.2021.e07344

**Published:** 2021-06-19

**Authors:** Ho Hoang Gia Bao, Hoang Phong Le

**Affiliations:** a School of Finance, University of Economics Ho Chi Minh City, 59C Nguyen Dinh Chieu, District 3, Ho Chi Minh City, Viet Nam; b School of Public Finance, University of Economics Ho Chi Minh City, 59C Nguyen Dinh Chieu, District 3, Ho Chi Minh City, Viet Nam

**Keywords:** Asymmetric effects, Nonlinear ARDL, Vehicle currency, J-curve, Trade balance, Vietnam, EU, UK

## Abstract

The relationship between exchange rate and trade balance has been spotlighted in the past several decades and thus examined by manifold research. The findings, however, lack of consensus despite the intensive efforts in investigating the role of exchange rate as an important determinant of trade balance in various countries. Although the existing papers are abundant, most of them neglect the role of vehicle currency. Besides, few articles are dedicated to Vietnam, and none has inspected the exchange rate-trade balance nexus between Vietnam and the EU. This study is the first to scrutinize how bilateral exchange rates, together with vehicle currency exchange rate, asymmetrically impact Vietnam's bilateral trade balance with respect to EU-27 countries and the UK. The NARDL estimation results strongly acknowledge the importance of USD as vehicle currency when more significant short-run and long-run coefficients are found. Accordingly, this article can provide some useful implications for policy-makers, especially when Vietnam was first labelled currency manipulator by the USA in December 2020. Particularly, USD/VND movement can affect not only Vietnam-USA but also Vietnam-EU and Vietnam-UK trade balance. In addition, VND appreciation against USD seems beneficial to Vietnam's bilateral trade with the EU plus the UK.

## 1 Introduction

The EU-Vietnam Free Trade Agreement (FTA) and Investment Protection Agreement (IPA) were signed in 2019 and the former came into force in August 2020, which can bring many benefits to both parties such as reducing trade barriers, boosting export and import, conserving EU agricultural products, fostering businesses, and protecting investment as well as the environment (European Commission, 2020a, European Commission, 2020b, European Commission, 2020c). In addition, the EU-Vietnam FTA is “the most comprehensive trade agreement the EU has concluded with a developing country”, which reveals that the EU appreciates the importance of Vietnam when she is the 17^th^ largest trading partner of the EU in total trade of goods as well as the 2^nd^ largest trading partner of the EU in the Association of South East Asian Nations (ASEAN) (European Commission, 2021). Also, the EU is the second-largest trading partner of Vietnam during the period 2000Q1–2018Q1^1^. Thus, the FTA and IPA agreements mark a notable milestone in economic integration and strategic partnership between the two parties. Moreover, they are expected to facilitate economic recovery and long-term growth of both parties after being negatively affected by the global COVID-19 pandemic (Vietnam's Ministry of Industry and Trade, 2020). Further, ASEAN is the third largest trading partner of the EU outside Europe, and it is very crucial for the EU to facilitate exportation to the 10 ASEAN countries by negotiating bilateral FTAs which serve as building blocks towards the future inter-regional EU-ASEAN trade agreement (European Commission, 2020a, European Commission, 2020b, European Commission, 2020c). Accordingly, after EU-Singapore FTA, the EU-Vietnam FTA coming into force in 2020 is considered the second step of the aforementioned process. Even though the UK now left the EU, the role of UK-Vietnam trade is not neglected. In fact, the UK-Vietnam FTA was signed in December 2020 and will officially come into effect in May 2021, signifying that Brexit does not change the continuity of economic integration as well as the importance of trade relationship between these countries. Hence, the trade between Vietnam and the EU-27 plus the UK is paid much attention and worth researching because useful recommendations can be made for policy-makers and other groups such as researchers, investors, business managers and stakeholders in not only Vietnam, the EU, and the UK but also other ASEAN members who expect to have free trade agreement with the EU and the UK in the future.

Export and import are at the core of international trade. The importance of export has been recognized in the export-led growth strategy aimed to boost economic growth in many countries and territories, for example, China, Hong Kong, India, Malaysia, Singapore, South Korea, Taiwan and Thailand (Tang et al., 2015); and Vietnam is not an exception. In addition, some countries in East and Southeast Asia (e.g., South Korea) supported their export-led growth strategy by keeping competitive exchange rate (Glasure and Lee, 1999; Gala, 2008) probably due to the association between economic growth and investment, international trade and currency depreciation (Hausmann et al., 2005). Besides the total export and import values, policy-makers as well as researchers also pay attention to the difference between them, i.e., trade balance. Trade balance can also be defined as the ratio of export to import (Bahmani-Oskooee, 1991), which in turns makes it convenient to depict the changes of export compared to import. Namely, when trade balance increases, export rises more than (or falls less than) import; and when trade balance decreases, export grows less than (or declines more than) import. Thus, trade balance is very helpful in demonstrating the direction of trade between a country and her partners.

Researching trade balance necessitates the scrutiny of its determinants, and exchange rate is presumably among the most crucial ones. The linkage between exchange rate-trade balance is a very popular topic in international finance and international economics disciplines. Traditionally, devaluation of domestic currency is believed to foster a country's trade balance, which motivates a large number of studies to test this belief so as to give implications for policy-makers to design effective policies to gain export advantage and foster trade balance. Early papers tried to validate the positive effect of devaluation on trade balance by checking the Marshall-Lerner condition: when the sum of import and export elasticities of demand in absolute terms surpasses 1, the reduction in value of domestic currency will boost trade balance (Bahmani-Oskooee and Niroomand, 1998; Bahmani-Oskooee and Mitra, 2010). Nevertheless, the Marshall-Lerner condition refers to the long-run impact of exchange rate on trade balance (Bahmani-Oskooee, 1991) and consequentially lacks the short-run counterpart. In fact, as trade balance needs some time to adjust under the influence of devaluation, short-run and long-run impacts of exchange rate could be distinguishable; and Magee (1973) proposed the term J-curve effect to depict the situation that devaluation can decrease trade balance in the short run and then encourage it in the long run. Since the introduction of J-curve effect, much more attention has been given to investigating the short-run and long-run impacts of exchange rate on trade balance (Bahmani-Oskooee and Mitra, 2010). Nonetheless, many research report insignificant results, and the existing literature has identified 2 main culprits: aggregation bias and linear assumption (Bahmani-Oskooee et al., 2016; Bahmani-Oskooee and Aftab, 2018; Iyke and Ho, 2018). First, although analyzing the impact of real effective exchange rate on the trade balance of a country with the rest of the world at aggregate level can be very convenient and present valuable information about her international trade as a whole, the findings may suffer from bias (Rose and Yellen, 1989; Bahmani-Oskooee and Brooks, 1999; Phong et al., 2018). Attempting to reduce the aggregation bias and search for more evidences of exchange rate's impacts on trade balance, researchers utilize data at bilateral and commodity levels in the trade between a country and each of her partners (e.g., Bahmani-Oskooee and Ratha, 2007; Bahmani-Oskooee and Hajilee, 2009). Second, even though disaggregating data can reduce aggregation bias, linear assumption (i.e., 1% depreciation and 1% appreciation of domestic currency have the same effect on trade balance) is another reason why a large number of studies could not detect cointegration and significant results (Bahmani-Oskooee and Fariditavana, 2016, 2020; Iyke and Ho, 2018). Therefore, recent papers well recognized the importance of asymmetric impacts of exchange rate on trade balance (Nusair, 2017; Iyke and Ho, 2017, 2018; Bahmani-Oskooee and Harvey, 2019; Bahmani-Oskooee and Kanitpong, 2019; Bahmani-Oskooee and Nasir, 2019; Hunter, 2019; Bahmani-Oskooee and Karamelikli, 2021; Bhat and Bhat, 2021). Nonetheless, besides the aforementioned culprits, in this paper we suspect that there may be still another culprit contributing to the insignificant findings of plentiful studies: the neglection of vehicle currency, i.e., the currency of the third country in the trade between the exporter and importer (Magee and Rao, 1980). Actually, vehicle currency has been ignored by the vast majority of the existing literature (Yang and Gu, 2016). This is a common weakness because it does not reflect reality that USD is the dominant vehicle currency in the world which appears on one side of nearly 87%–88% of the global trade (Krugman, 1980; McKinnon, 2002; Devereux et al., 2007; Bank for International Settlements, 2016, 2019). Moreover, USD has been employed in more than 200 countries and territories (Liu et al., 2019), and thus it is frequently utilized in the trade of many countries with their trading partners which are different from the USA (Boz et al., 2020). Despite the vital importance of USD in the international trade, nearly all bilateral-level^2^ and commodity-level research overlook the role of USD as vehicle currency. The disregard for the role of vehicle currency is unfavorable for the research on exchange rate-trade balance nexus in developing countries that heavily utilize USD as vehicle currency because it fails to capture the reality of trade context and thus lacks crucial findings necessary for policy implications, which can be deemed a research gap. Realizing the importance of USD, this paper incorporates its role as vehicle currency to scrutinize the exchange rate-trade balance linkage for the case of Vietnam. Also, recognizing the drawback of linear assumption, this paper employs NARDL method to evaluate the asymmetric impacts of bilateral exchange rates as well as vehicle currency exchange rate on Vietnam's bilateral trade balance with the EU-27 and the UK. The empirical results strongly support the essential role of USD as vehicle currency in Vietnam-EU and Vietnam-UK trade.

The findings of this paper are very compatible with the reality that about 90% of Vietnam's international trade in 2016 was denominated in USD, while the shares of EUR, GBP, and JPY were only 4.5%, 1.2%, and 1.0% respectively (Agency of Vietnam's Ministry of Planning and Investment, 2017; Agency of Vietnam's Ministry of Finance, 2018). Also, concerning the total trade between EU-27 countries and the UK with countries outside the EU from 2010 to 2018, the share of USD was always higher than that of EUR (Eurostat, 2021), which affirms that USD has been a crucial vehicle currency in this region despite the presence of the world's second most traded currency (i.e., EUR) which is the official currency of many EU members (Bank for International Settlements, 2016, 2019). Consequently, USD serves as a key vehicle currency in the trade between Vietnam and EU-27 countries plus the UK, and thus it cannot be neglected. Besides, the findings of this study can provide useful information for policy-makers, especially in the circumstance that Vietnam was regarded as currency manipulator by the USA in December 2020 for the first time in history (Mohsin and McCormick, 2020). If increasing the value of VND against USD is applied as one of the solutions to the currency manipulator status, not only Vietnam-USA trade balance can be affected but Vietnam-EU and Vietnam-UK ones can also be influenced because USD is highly used as vehicle currency in the trade between Vietnam and the involved European partners. Hence, the noticeable difference between the impacts of bilateral exchange rates and vehicle currency exchange rate on Vietnam's trade balance with respect to the EU-27 plus the UK is that the latter is strongly correlated with USD/VND exchange rate movement which is more strongly influenced by the Vietnam-USA relationship in terms of trade, economic, diplomatic and other aspects. Thus, different invoicing currencies can affect trade balance due to different exchange rate fluctuations and pass-through (Gopinath et al., 2010; Eurostat, 2020; Boz et al., 2020).

The main objective of this paper is to evaluate the asymmetric impacts of bilateral exchange rates as well as vehicle currency exchange rate on Vietnam's trade balance with each of the EU-27 countries and the UK.

This paper provides some notable contributions. Firstly, it is worth researching in the current trade context of Vietnam. Specifically, the EU-Vietnam FTA was effective in August 2020, and not long after Brexit, the UK-Vietnam FTA was signed in December 2020 and will come into force in May 2021, which reflects the reciprocal importance of Vietnam's trade with the EU-27 as well as the UK. Also, the aforesaid free trade agreements profoundly strengthen the economic integration between Vietnam and the involved parties. In addition, the fact that Vietnam was first labelled currency manipulator by the USA in December 2020 (Mohsin and McCormick, 2020) attracts more attention to the foreign exchange management of Vietnam in general and the USD/VND exchange rate in particular. With the heavy employment of USD in around 90% of Vietnam's total trade value, the change of USD/VND could affect not only Vietnam's trade balance with the USA but also with the EU and the UK. Thus, this paper captures the reality that USD is a dominant vehicle currency in Vietnam's trade with the EU and the UK, which gives useful information for Vietnam's policy-makers in designing and implementing effective foreign exchange as well as trade policies. Secondly, to the best knowledge of the authors, no research has analyzed the influences of bilateral exchange rates on Vietnam's trade balance with each of EU-27 countries and the UK. In addition, no research has inspected the role of vehicle currency in Vietnam-EU and Vietnam-UK trade. Hence, this is the first study to cover these issues. Moreover, this study can avoid linear assumption by using NARDL method and thus can capture the impacts of exchange rates on Vietnam's trade balance more effectively than the conventional ARDL model. Furthermore, this paper can compare the world's two leading currencies (i.e., USD and EUR) in validating the J-curve phenomenon as well as the Marshall-Lerner condition for Vietnam in the trade with the selected European partners.

The subsequent content of this study follows a standard structure: Part 2 provides literature review; Part 3 depicts the data and method; Part 4 demonstrates empirical results; and Part 5 is the conclusion.

## 2 Literature review

The Marshall-Lerner condition stipulates that a country's trade balance is positively affected by the depreciation of her currency when the absolute export demand elasticity plus the import counterpart is bigger than 1 (Bahmani et al., 2013). Magee (1973) documented that the aforesaid impact can manifest after some period of time during which the trade balance declines and thus proposed the term “J-curve” effect. Since then, manifold studies have devoted to examine the J-curve effect as well as the link between exchange rate and trade balance in various countries and reported different findings (Bahmani-Oskooee and Ratha, 2004; Bahmani-Oskooee and Hegerty, 2010; Phong et al., 2019a).

The vast majority of J-curve-related research implies symmetric connection between exchange and trade balance (Bahmani-Oskooee and Aftab, 2017). This group of studies has employed many different methods to inspect J-curve phenomenon. Early articles on the relationship between exchange rate and trade balance utilized the natural logarithm of level variables and suffered from non-stationarity problem (Rose and Yellen, 1989). Bahmani-Oskooee (1985) was the first research to introduce the method for testing J-curve effect by allowing some lags of exchange rate and then observing the change of signs. Rose and Yellen (1989) employed Engle-Granger cointegration technique and proposed a new way to detect J-curve effect: negative short-run coefficients of exchange rate combined with positive long-run ones. They found no J-curve phenomenon in the trade between the USA and Canada, France, Germany, Italy, Japan, and the UK. Other studies applied Johansen cointegration technique, for example, Wilson (2001), Lal and Lowinger (2002), Hacker and Hatemi-J (2004), and Yusoff (2007). Besides, Autoregressive Distributed Lag (ARDL) has become one of the most utilized methods for identifying J-curve effect in the literature (Bahmani-Oskooee and Ratha, 2004; Bahmani-Oskooee and Hegerty, 2010). Studies employing ARDL can be instanced as Arora et al. (2003), Bahmani-Oskooee and Ratha (2007), Duasa (2007), Kyophilavong et al. (2013), Durmaz (2015), and Phong et al. (2018).

Thanks to the introduction of Nonlinear Autoregressive Distributed Lag (NARDL) model by Shin et al. (2014), researchers have the tool to differentiate the impact of domestic currency depreciation on trade balance from appreciation. Bank for International Settlements, 2019, Bahmani-Oskooee and Fariditavana, 2016, Bahmani-Oskooee and Fariditavana, 2015 as well as Nusair (2017) questioned if the failure in detecting J-curve phenomenon was due to the linear assumption of exchange rate-trade balance nexus. They proved that applying NARDL method could help identify more evidences. Likewise, Iyke and Ho (2017) detected J-curve effect for the case of Ghana when employing NARDL method on aggregate-level data during 1986Q1–2016Q3, which is not found when symmetric assumption and ARDL approach are used. Also, Iyke and Ho (2018) inspected the bilateral trade of South Africa with respect to China, Germany, India, Japan, the UK and the USA from 1998Q1 to 2016Q2. They applied both ARDL and NARDL methods: while the former helped identify J-curve effect associated with India and the UK, the latter was able to disclose J-curve effect connected with all trading partners. Thus, their findings strongly indicate that NARDL is more superior than the conventional ARDL method in providing support for J-curve phenomenon. Many other papers reported the same results, which can be instanced as Bahmani-Oskooee and Baek (2018), Bahmani-Oskooee et al., 2018a, Bahmani-Oskooee et al., 2018b, Ari et al. (2019), Bahmani-Oskooee and Fariditavana, 2020, Bahmani-Oskooee et al., 2019a, Bahmani-Oskooee et al., 2019b, Bahmani-Oskooee and Nasir (2019), Bahmani-Oskooee and Fariditavana (2020), and Bahmani-Oskooee and Nouira (2020). Consequently, there is a firm basis for the asymmetrical influence of exchange rate on trade balance, and NARDL is more appropriate for examining J-curve hypothesis than the linear ARDL method.

While the existing literature about J-curve effect in the trade between European Union (EU) countries and their partners is plentiful (e.g., Hacker and Hatemi-J, 2003; 2004; Bahmani-Oskooee et al., 2006; Hsing, 2009; Hsing and Sergi, 2009; Šimáková and Stavárek, 2013; 2014; Nusair, 2017; Michail, 2018; Gürtler, 2018; Lucarelli et al., 2018), few studies are dedicated for Vietnam. Phan and Jeong (2015) analyzed Vietnam's trade balance with 16 partners by using Fully Modified OLS and Dynamic OLS estimators for the panel data ranging from 1999Q1 to 2012Q4 and reported that exchange rate negatively influenced trade balance and thus no J-curve effect. Lee (2018), utilizing ARDL approach, observed no J-curve at aggregate level (i.e., Vietnam's trade with the rest of the world represented by 15 largest partners) and bilateral level (i.e., Vietnam's trade with each of the 15 largest partners) in the period 1996Q1–2016Q4. Lee (2020) used Fully Modified OLS estimator on annual data from 1994 to 2016 and indicated that the Marshall-Lerner condition was not satisfied in the trade between Vietnam and the majority of trading partners. On the contrary, Phong et al. (2018) employed ARDL technique to scrutinize the impact of real effective exchange rate on Vietnam's trade balance with respect to the rest of the world proxied by 22 largest trading partners in the period 2000Q1–2015Q4 and found evidence of J-curve effect and thus validating the Marshall-Lerner condition in Vietnam at aggregate level. The aforementioned studies assumed linear relationship between Vietnam's exchange rate and trade balance, which possibly neglects the asymmetrical impact of exchange rate on trade balance in the short run and long run. Hence, Phong et al., 2019a, Phong et al., 2019b applied NARDL method to inspect the influence of real effective exchange rate on Vietnam's trade balance at aggregate level with 26 largest trading partners representing the rest of the world during 2000Q1–2018Q1 to explore if depreciation and appreciation of VND had distinguishable effects. They discovered that both the depreciation and appreciation of VND facilitated Vietnam's trade balance in the long run, and the magnitude of impact of the former is bigger than the latter, which supported asymmetric long-run effects as well as the Marshall-Lerner condition in Vietnam. Moreover, they showed that the asymmetric impact of real effective exchange rate also occurred in the short run.

## 3 Data and methodology

A large body of literature employs the standard two-country model to analyze the impact of exchange rate on trade balance (Rose and Yellen, 1989; Hsing and Sergi, 2009; Iyke and Ho, 2017, 2018; Phong et al., 2018; Bahmani-Oskooee and Nasir, 2019; Bahmani-Oskooee and Nouira; 2020), which is depicted in natural logarithm form as follows:

(1) $$lnTB_{i,t}=\alpha_{i}+\beta_{i}.lnBER_{i,t}+\gamma_{i}.lnGDP_{t}+\delta_{i}.lnGDPF_{i,t}+\varepsilon_{i,t}$$

In Eq. (1), $TB_{i,t}$ denotes the trade balance (the ratio export/import) of Vietnam with respect to partner i at time t; $BER_{i,t}$ represents the bilateral real exchange rate between the currency of partner i and VND at time t (an increase of this variable signifies the depreciation of VND); $GDP_{t}$ indicates the real income of Vietnam at time t; $GDPF_{i,t}$ stands for the real income of partner i at time t; and $\varepsilon_{i,t}$ is the error term. The data frequency is quarterly, ranging from 2000Q1 to 2018Q1. All variables are converted into indices with the value of the base period 2000Q1 is 100. The data comes from various sources including Asian Development Bank (ADB), Eurostat (downloaded from the website of Federal Reserve Bank of St. Louis (FRED)), General Statistics Office of Vietnam (GSO), International Monetary Fund's (IMF) International Financial Statistics (IFS) and Direction of Trade Statistics (DOTS).

So as to scrutinize the role of USD as the vehicle currency in the trade between Vietnam and EU-27 countries plus the UK, we replace the bilateral exchange rate by the USD/VND exchange rate:

(2) $$lnTB_{i,t}=\alpha \prime_{i}+\text{β}{\text{′}}_{i}.lnUSD_{t}+\text{γ}{\text{′}}_{i}.lnGDP_{t}+\delta \prime_{i}.lnGDPF_{i,t}+\varepsilon \prime_{i,t}$$

where $USD_{t}$ means the real exchange rate USD/VND at time t, and an increase of USD/VND is associated with the depreciation of VND.

In this paper, NARDL method proposed by Shin et al. (2014) is employed because of its advantages and suitability. First, it allows evaluating the asymmetric impacts of independent variables on dependent variable. Thus, it helps examine how exchange rate asymmetrically affects trade balance in the case of Vietnam. Second, the recent literature well documented that exchange rate has asymmetric influences on trade balance in many countries, and the application of NARDL model can provide more significant and detailed findings than the linear ARDL approach (Bahmani-Oskooee and Fariditavana, 2015; 2016; 2020; Bahmani-Oskooee and Aftab, 2017; Bahmani-Oskooee and Baek, 2018; Iyke and Ho, 2017; 2018; Bahmani-Oskooee et al., 2019a, Bahmani-Oskooee et al., 2019b; Bahmani-Oskooee and Nouira, 2020). Third, failing to capture the asymmetric influences of exchange rate on trade balance is one reason why papers employing ARDL model as well as assuming linear impacts of exchange rate could not detect cointegration and significant results (Bahmani-Oskooee and Fariditavana, 2016; Bahmani-Oskooee and Fariditavana, 2020; Iyke and Ho, 2018). Hence, NARDL method is employed by manifold recent studies (e.g., Nusair, 2017; Iyke and Ho, 2017; 2018; Bahmani-Oskooee and Harvey, 2019; Bahmani-Oskooee and Kanitpong, 2019; Bahmani-Oskooee and Nasir, 2019; Hunter, 2019; Bahmani-Oskooee et al., 2021; Bahmani-Oskooee and Karamelikli, 2021; Bhat and Bhat, 2021; Usman et al., 2021). Fourth, NARDL has all strengths of ARDL. Namely, it permits the combination of I(0) and I(1) variables (Pesaran et al., 2001; Shin et al., 2014; Bahmani-Oskooee and Baek, 2018; Bahmani-Oskooee and Nasir, 2019). This is one of the most notable advantages of NARDL and ARDL models compared to traditional cointegration methods such as Johansen (1991) and Engle and Granger (1987) because they require that all variables must be I(1) processes. As a result, unit root tests are unnecessary because most of the macroeconomic variables are integrated of order 1 (Bahmani-Oskooee et al., 2017; Bahmani-Oskooee and Aftab, 2018; Bahmani-Oskooee and Baek, 2018; Iyke and Ho, 2018; Bahmani-Oskooee and Nasir, 2019). In addition, ARDL method is still appropriate despite small sample size (Odhiambo, 2009). Furthermore, it can still provide unbiased estimates when some variables are endogenous (Phong et al., 2018). Fifth, the ARDL approach can effectively assess the short-run reaction of trade balance under the influence of exchange rate (Iyke and Ho, 2018). Last but not least, because many trading partners’ models in our study have both I(1) and I(0) variables, the usage of NARDL method is appropriate.

In order to evaluate the asymmetric short-term and long-term effects of exchange rates on trade balance, we transform Eqs. (1) and (2) into error correction form, indicated by Eqs. (3) and (5) respectively, following Pesaran et al. (2001) and Shin et al. (2014):

(3) $$\text{Δ}lnTB_{i,t}={\text{ω}}_{i}+\overset{p_{1}}{\underset{j=1}{\sum }}\left(\alpha_{i,j}.\text{Δ}lnTB_{i,t-j}\right)+\overset{p_{2}}{\underset{k=0}{\sum }}\left(\beta_{i,k}^{+}.\text{Δ}POS_{i,t-k}\right)+\overset{p_{3}}{\underset{l=0}{\sum }}\left(\beta_{i,l}^{-}.\text{Δ}NEG_{i,t-l}\right)+\overset{p_{4}}{\underset{m=0}{\sum }}\left(\gamma_{i,m}.\text{Δ}GDP_{t-m}\right)+\overset{p_{5}}{\underset{n=0}{\sum }}\left(\delta_{i,n}.\text{Δ}lnGDPF_{i,t-n}\right)+\lambda_{1,i}.lnTB_{i,t-1}+\lambda_{2,i}.POS_{i,t-1}+\lambda_{3,i}.NEG_{i,t-1}+\lambda_{4,i}.lnGDP_{t-1}+\lambda_{5,i}.lnGDPF_{i,t-1}+e_{i,t}$$

In Eq. (3), $POS_{i}$ and $NEG_{i}$ respectively denote the partial sum of positive and negative changes in bilateral real exchange rate with respect to partner i. According to Shin et al. (2014), we calculate them as:

(4) $$POS_{i,t}=\overset{t}{\underset{r=1}{\sum }}\text{max}\left(\text{Δ}lnBER_{i,r},0\right);NEG_{i,t}=\overset{t}{\underset{r=1}{\sum }}\text{min}\left(\text{Δ}lnBER_{i,r},0\right)$$

Eq. (5) is used for analyzing how the real exchange rate between the vehicle currency USD and VND influences Vietnam's trade balance with each partner in the short run as well as the long run:

(5) $$\text{Δ}lnTB_{i,t}=\text{ω}{\text{′}}_{i}+\overset{q_{1}}{\underset{j=1}{\sum }}\left(\alpha \prime_{i,j}.\text{Δ}lnTB_{i,t-j}\right)+\overset{q_{2}}{\underset{k=0}{\sum }}\left(\beta \prime_{i,k}^{+}.\text{Δ}POS_{t-k}\right)+\overset{q_{3}}{\underset{l=0}{\sum }}\left(\beta \prime_{i,l}^{-}.\text{Δ}NEG_{t-l}\right)+\overset{q_{4}}{\underset{m=0}{\sum }}\left(\delta \prime_{i,m}.\text{Δ}GDP_{t-m}\right)+\overset{q_{5}}{\underset{n=0}{\sum }}\left(\delta \prime_{i,n}.\text{Δ}lnGDPF_{i,t-n}\right)+\lambda \prime_{1,i}.lnTB_{i,t-1}+\lambda \prime_{2,i}.POS_{i,t-1}+\lambda \prime_{3,i}.NEG_{i,t-1}+\lambda \prime_{4,i}.lnGDP_{t-1}+\lambda \prime_{5,i}.lnGDPF_{i,t-1}+e\prime_{i,t}$$

where POS and NEG represent the partial sum of positive and negative changes in USD/VND real exchange rate. The definition of POS and NEG is described as:

(6) $$POS_{t}=\overset{t}{\underset{r=1}{\sum }}\text{max}\left(\text{Δ}lnUSD_{r},0\right);\;NEG_{t}=\overset{t}{\underset{r=1}{\sum }}\text{min}\left(\text{Δ}lnUSD_{r},0\right)$$

The J-curve effect can be identified if the short-run coefficients of either POS or NEG variables in Eqs. (3) and (5) are negative or statistically insignificant while their long-run counterparts are positive (Rose and Yellen, 1989; Bahmani-Oskooee and Fariditavana, 2015; Bahmani-Oskooee et al., 2018a, Bahmani-Oskooee et al., 2018b).

Eq. (3) can be estimated by the standard procedure of ARDL model (Shin et al., 2014). Thus, first, it is necessary to ensure that the order of integration of each variable^3^ is less than 2 (Pesaran et al., 2001; Phong et al., 2019b). Second, the bound test for cointegration needs to be conducted (Pesaran et al., 2001). For example, regarding Eq. (3), the null hypothesis of the bound test for the i^th^ partner is no long-run effect (H_0_: $\lambda_{1,i}=\lambda_{2,i}=\lambda_{3,i}=\lambda_{4,i}=\lambda_{5,i}=0$) and the alternative hypothesis is the occurrence of long-run effect (H_1_: $\lambda_{1,i}\neq \lambda_{2,i}\neq \lambda_{3,i}\neq \lambda_{4,i}\neq \lambda_{5,i}\neq 0$). Third, after the cointegration among the variables is validated, estimation for short-run and long-run coefficients can be implemented. Finally, the stability and reliability of the model can be checked by Cumulative Sum of Recursive Residuals (CUSUM), Cumulative Sum of Square of Recursive Residuals (CUSUMSQ), Breusch-Godfrey, Breusch–Pagan and Ramsey RESET tests. The estimation process for Eq. (5) is similar to the aforementioned steps.

## 4 Empirical results

The NARDL estimation of the two models: bilateral exchange rate (denoted as BER, specified in Eq. (3)) and vehicle currency exchange rate (denoted as USD, specified in Eq. (5)) is indicated in the Appendix.

The cases with J-curve effect are summarized in Table 1. Regarding the bilateral exchange rate model, the J-curve effect caused by POS (i.e., depreciation of VND) is witnessed in the cases of France, Greece, Portugal, Romania, Spain and Sweden, as evidenced by the significant negative or insignificant short-run coefficients of POS variables combined with their corresponding positive long-run ones (Rose and Yellen, 1989; Bahmani-Oskooee and Fariditavana, 2015; Bahmani-Oskooee et al., 2018a, Bahmani-Oskooee et al., 2018b). Among the aforementioned countries, France is the third biggest EU trading partner of Vietnam during the 2000Q1–2018Q1 period. Based on the NARDL estimation outcome, 1% depreciation of VND against EUR hurts Vietnam's trade balance with France in the short run and then improves it in the long run by approximately 1.11%. Remarkably, 1% appreciation of VND (indicated by NEG variable) boosts Vietnam's trade balance with France in the short run before decreasing it by nearly 0.39% in the long run. Hence, the impacts of depreciation and appreciation of VND against EUR in the trade between Vietnam and France contradict each other in both short run and long run, which is very similar to the case of Romania.

**Table 1:** Summary of J-curve effect in the trade between Vietnam and EU-27 and the UK.

Model	J-curve effect cases
BER	France, Greece, Portugal, Romania, Spain, Sweden
USD	Austria, France, Latvia, UK

Regarding the vehicle currency model, the J-curve effect caused by VND depreciation against USD (POS) is detected in the cases of Austria, France, Latvia and the UK. Remarkably, the trade balance between Vietnam and France also experiences J-curve effect in the vehicle currency model. Both EUR/VND and USD/VND exchange rates are important in Vietnam-France trading relationship, and the use of USD as vehicle currency may be beneficial to Vietnam when the depreciation as well as appreciation of VND against USD facilitate her trade balance in the long run.

**Figure 1:**
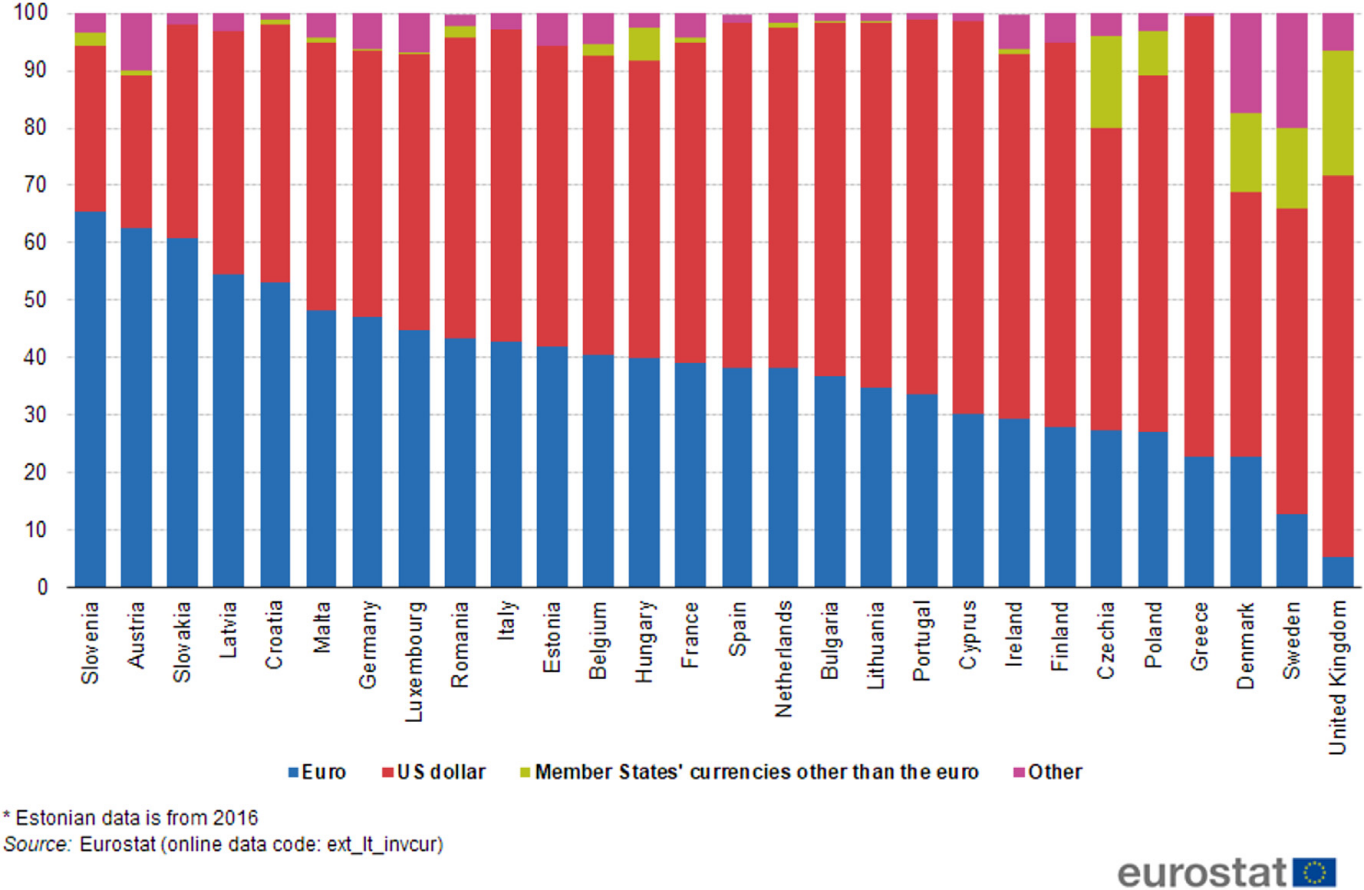
Extra-EU imports of goods by invoicing currency (%).

**Figure 2:**
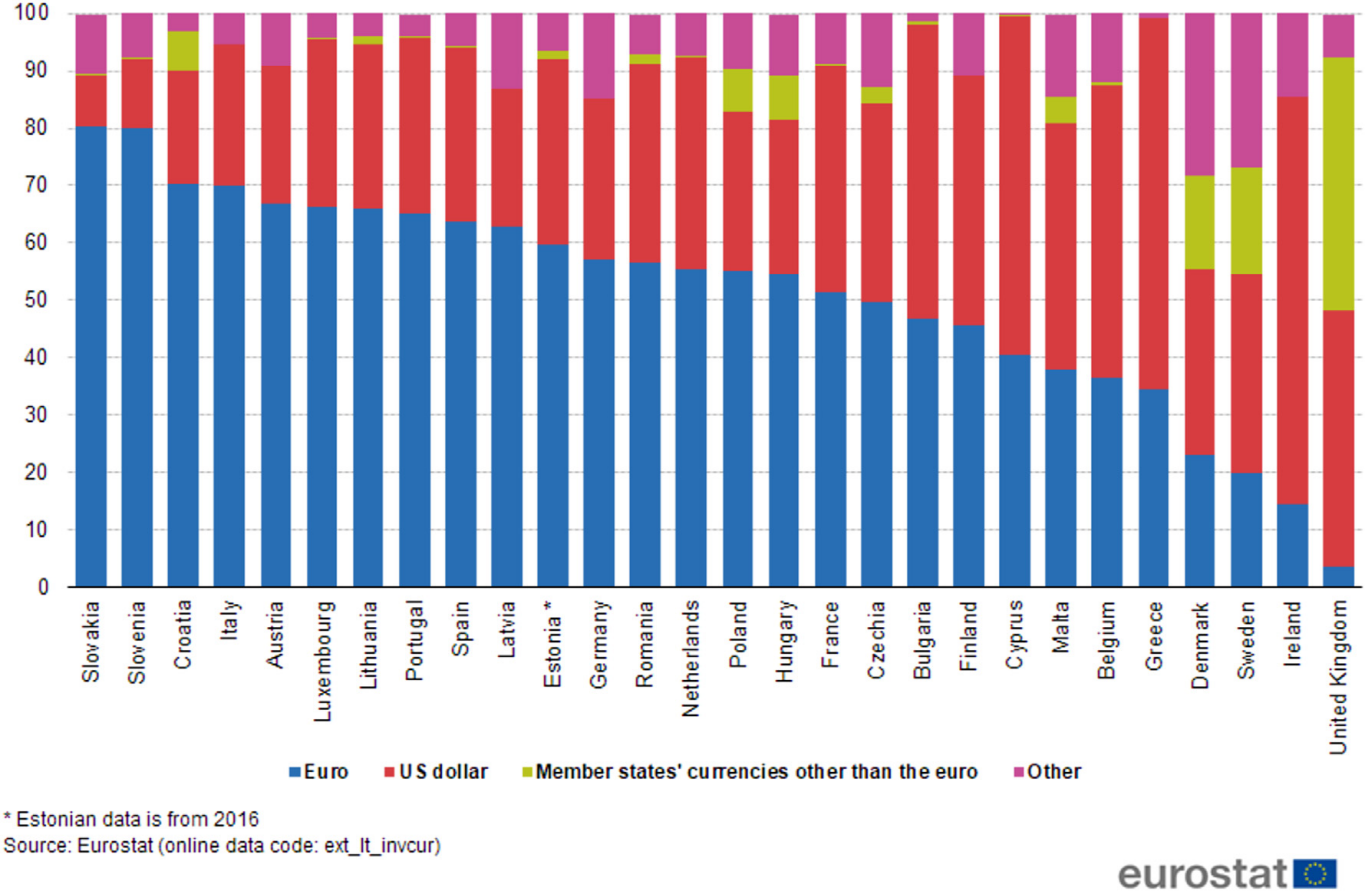
Extra-EU exports of goods by invoicing currency (%).

Another notable trading partner of Vietnam is the UK, the second largest economy in Europe and also the second largest European trading partner of Vietnam in the period 2000Q1–2018Q1. In addition, the UK possesses the strong currency GBP which is the fourth most traded currency in the world (Bank for International Settlements, 2019). While Vietnam's trade balance deteriorates under the influence of GBP/VND in the short run, it is unresponsive in the long run. Nonetheless, the depreciation of VND against USD induces J-curve effect on Vietnam's trade balance, which may be advantageous to Vietnam when USD is much employed as vehicle currency in trading with the UK. This argument is supported by the data in Figure 1 and Figure 2 that USD is utilized more than GBP in the importation of the UK from countries outside the EU, combined with the fact that Vietnam experiences trade surplus with the UK during 2000Q1–2018Q1, and approximately 90% of Vietnam's international trade value is invoiced in USD. Accordingly, it can be implied that although the bargaining power of the UK is possibly higher than Vietnam and may consequentially affect the choice of GBP as invoicing currency, USD is still the important vehicle currency in the trade between these two countries, probably due to the nature of exported and imported commodities (Magee and Rao, 1980). Future studies can investigate the Vietnam-UK trade at commodity level to further compare the role of USD and GBP. Besides, Vietnam's and the UK's incomes significantly stimulate the exportation of both countries: when Vietnam's real GDP increases by 1%, her trade balance is lowered by about 5.4% as the exportation of the UK rises; and when the UK's real GDP goes up by 1%, it encourages Vietnam's trade balance by nearly 9.3% as the exportation of Vietnam is enhanced.

Germany, the largest economy in Europe and also the largest European trading partner of Vietnam in the period 2000Q1–2018Q1, is a member of the euro area and thus utilizes EUR as her official currency. From the results in Table 1, when VND depreciates against EUR or USD, Vietnam's trade balance in the short run and long run is negatively affected, and the impact magnitude of USD/VND is stronger. This outcome may imply that both EUR and USD are substantially used in the bilateral trade between Vietnam and Germany. Indeed, it can be observed in Figures 1 and 2 that USD holds a considerable share in the trade, especially importation, of Germany with partners outside the EU. Furthermore, roughly 90% of Vietnam's international trade is denominated in USD and Vietnam has trade surplus with Germany in the sampled period. Hence, the above statistics can reinforce that USD is a crucial vehicle currency in Vietnam-Germany trade. And the estimation results indicate that the depreciation of VND against USD hurts Vietnam's trade balance with Germany. Moreover, the appreciation of VND against USD exhibits no impact. Consequently, Vietnam's trade balance with Germany cannot be encouraged by exchange rate. Nevertheless, it can be facilitated mainly by Germany's income because the sign of the variable GDPF in the vehicle currency model is positive and significant with relatively large value.

The findings of our study can be discussed in comparison with those of Phan and Jeong (2015) and Lee (2018). First, regarding the trade between Vietnam and Germany, Phan and Jeong (2015) reported that real exchange rate positively affected Vietnam's trade balance in the period 1999Q1–2012Q4. However, our results show that both appreciation and depreciation of bilateral exchange rate decrease trade balance in the long run, which is similar to Lee (2018). In addition, we also discover that USD/VND appreciation hurts trade balance in the long run at a greater magnitude. Second, concerning Vietnam-UK trade, Phan and Jeong (2015) found different results depending on different estimators; namely, when FMOLS is used, GBP/VND enhances trade balance, but the impact is positively insignificant in case of DOLS. Meanwhile, Lee (2018) utilized ARDL technique and indicated the encouraging effect of GBP/VND on Vietnam's trade balance between 1996Q1 and 2016Q4. In this study, we also observe positive but insignificant coefficients of GBP/VND appreciation as well as depreciation in the long run by employing NARDL method. Besides, we demonstrate that both USD/VND appreciation and depreciation facilitate Vietnam's trade balance in the long run. This, therefore, sheds new light on the topics regarding the vehicle currency and J-curve effect for the trade between Vietnam and the UK. Third, while Lee (2018) did not include France in the sample, Phan and Jeong (2015) reported inconclusive results: no effect of exchange rate on trade balance in the FMOLS model but negative impact in the DOLS one. In contrast, with the application of NARDL method, we detect J-curve effect of EUR/VND as well as USD/VND on Vietnam's trade balance, and at the same time, we can distinguish the influences of VND depreciation from VND appreciation. Hence, our study explores new findings and contributes to the literature on exchange rate-trade balance nexus by incorporating the role of USD as the important vehicle currency along with the usage of NARDL model.

Table 2 provides evidence for the usefulness of scrutinizing the role of USD when analyzing Vietnam's bilateral trade balance with EU-27 countries and the UK, which can be attributed to the reality that USD is the dominant currency used in Vietnam's international trade. Namely, 90% of Vietnam's international trade value was invoiced in USD, while the shares of EUR, GBP, and JPY were only 4.5%, 1.2%, and 1.0% respectively (Agency of Vietnam's Ministry of Planning and Investment, 2017; Agency of Vietnam's Ministry of Finance, 2018). Moreover, based on the Direction of Trade Statistics data provided by IMF, in 2016, the euro area (i.e., 19 EU Member States using EUR as their official currency) occupied more than 9.1% the total trade value of Vietnam, which was around twice as big as the percentage of Vietnam's trade value invoiced in EUR (i.e., 4.5%). This indicates that, besides the 4.5% portion, the remaining trade value between Vietnam and the euro area (i.e., 4.6%) had to be settled by other currencies, and it was very likely the case that USD was the vehicle currency. Furthermore, in 2016, the EU was the third-largest trading partner of Vietnam, holding about 12% of Vietnam's total trade value. Nevertheless, the currencies of the EU were not used much in the trade with Vietnam, thus signifying the important role of USD as vehicle currency. This is strongly reinforced by the data denoting that while 90% of Vietnam's trade value was denominated in USD, the share of the USA in Vietnam's total trade was only 13.2%^1^. Analogous to the above-mentioned statistics and arguments, the results in Table 2 once again acknowledge the importance of USD as vehicle currency in Vietnam-EU trade when the USD models have more significant short-run and long-run coefficients than the bilateral models. Moreover, additional cases satisfying the J-curve effect and Marshall-Lerner condition are detected in Tables 1 and 3 thanks to the incorporation of USD into the analyses of Vietnam's bilateral trade with the EU-27 and the UK.

**Table 2:** Number of cases with significant long-run and short-run coefficients (POS and NEG) in BER and USD models.

Model	Long-run coefficients	Short-run coefficients	Bothlong-run and short-run coefficients
BER	22	26	21
USD	23	27	23

In other to check for the validity of Marshall-Lerner condition in Vietnam, we summarize the long-run effects of VND depreciation in both BER and USD models in Table 3. When domestic currency depreciation positively impacts trade balance in the long run, the existence of Marshall-Lerner condition is implied (Rose, 1991; Bahmani-Oskooee, 1991; Bahmani-Oskooee and Wang, 2006; Bahmani-Oskooee and Kanitpong, 2019). Thus, in BER model, the Marshall-Lerner condition is supported in the trade between Vietnam and 9 countries including Austria, Cyprus, Denmark, France, Greece, Portugal, Romania, Spain and Sweden when VND depreciation against their currencies fosters Vietnam's trade balance. Additionally, the Marshall-Lerner condition is also validated when VND depreciation against the vehicle currency USD improves Vietnam's trade balance in the trade with Austria, Cyprus, France, Greece, Latvia, Poland and the UK. Remarkably, the cases of 4 partners Austria, Cyprus, France and Greece demonstrate robust evidences when VND depreciation against EUR or USD boosts Vietnam's trade balance in the long run, thus reinforces the proof of Marshall-Lerner condition. Moreover, the Marshall-Lerner condition is found in the trade between Vietnam and the UK and France (respectively the 2^nd^ and 3^rd^ largest European partners of Vietnam), along with the occurrence of J-curve effect. Our findings, therefore, are different from Lee (2020) which concluded no Marshall-Lerner condition in the trade between Vietnam and France as well as the UK. The culprits for this discrepancy might be that Lee (2020) used different time frame, data frequency and method, assumed linear impacts of bilateral exchange rate and did not cover the role of USD as vehicle currency. Our study can find evidence for the Marshall-Lerner condition in the Vietnam-France and Vietnam-UK trade by analyzing the nonlinear impacts of both bilateral and vehicle currency exchange rates. This indicates the usefulness of including the role of vehicle currency in the inspection of exchange rate-trade balance linkage.

**Table 3:** The summary of long-run effects of VND depreciation and appreciation on trade balance.

	BER model	USD model
VND depreciation encourages trade balance	Austria, Cyprus, Denmark, France, Greece, Portugal, Romania, Spain, Sweden	Austria, Cyprus, France, Greece, Latvia, Poland, UK
VND appreciation encourages trade balance	Croatia, Cyprus, Denmark, Greece, Lithuania, Slovenia, Spain	Austria, Croatia, Cyprus, Finland, France, Greece, Ireland, Malta, Romania, Slovakia, Slovenia, Spain, Sweden, UK
VND depreciation reduces trade balance	Bulgaria, Czechia, Germany, Luxembourg, Poland, Slovakia	Bulgaria, Germany, Ireland, Lithuania, Portugal
VND appreciation reduces trade balance	Belgium, Estonia, France, Germany, Hungary, Luxembourg, Netherlands, Romania, Slovakia	Belgium, Bulgaria, Hungary, Latvia, Lithuania, Luxembourg, Portugal

The results presented in Table 3 also summarize the influences of bilateral exchange rates and USD/VND exchange rate on Vietnam's bilateral trade balance with countries from the EU-27 and the UK, which can provide helpful information especially when Vietnam was labelled currency manipulator by the USA in December 2020 for the first time in history. Relating to this incident, Vietnam was criticized by the US for devaluating VND against USD by more than 8% in recent years to gain export competitiveness although Vietnam disagreed with that claim (Mohsin and McCormick, 2020; Lynch, 2020). In addition, Vietnam met all the criteria of a currency manipulator according to the USA: current account surplus of 2% GDP, trade balance surplus of $20 billion USD, and foreign exchange interventions of 2% GDP (Mohsin and McCormick, 2020). The negotiation between Vietnam and the US is ongoing and actual actions of both parties to solve the situation are still ahead. For example, the USA may demand that Vietnam should reduce foreign exchange interventions and alter the foreign exchange management to let VND appreciate against USD more quickly. This adjustment process can impact the USD/VND exchange rate, which in turns affects not only Vietnam's trade with the USA but also with the EU-27 and the UK as well as other large trading partners such as China because USD is heavily employed as vehicle currency. Table 3 indicates that, when VND appreciates against USD, Vietnam's bilateral trade balance with 7 EU countries (i.e., Belgium, Bulgaria, Hungary, Latvia, Lithuania, Luxembourg and Portugal) is badly affected. The aforesaid 7 countries altogether held only 8.1%^1^ of the total trade value between Vietnam and the EU in the period 2000Q1–2018Q1. Hence, the negative impact is not very considerable. In contrast, the appreciation of VND against USD can boost Vietnam's trade balance with 14 countries (i.e., Austria, Croatia, Cyprus, Finland, France, Greece, Ireland, Malta, Romania, Slovakia, Slovenia, Spain, Sweden and the UK) which constituted more than 43.1% of Vietnam's total trade value with the EU in the period 2000Q1–2018Q1. As a result, the appreciation of VND against USD possibly brings more benefit than harm to Vietnam-EU trade. In the process of solving the currency manipulator status, if VND appreciates against USD more quickly, Vietnam's trade balance with the EU-27 and the UK can be fostered.

## 5 Conclusion

This study employs NARDL method to investigate the asymmetric impacts of bilateral as well as vehicle currency exchange rates on Vietnam's trade balance with EU-27 countries and the UK in the period 2000Q1–2018Q1. The estimation results indicate strong support for the vital role of USD as vehicle currency, even when the partners are mainly from the EU where EUR, the world's second most traded currency, is the official currencies of 19 out of 27 members. With the presence of USD, more significant short-run and long-run asymmetric impacts are found. Remarkably, France is the partner that Vietnam experiences J-curve effect of her trade balance induced by both EUR/VND and USD/VND. Another noticeable result is that GBP/VND does not influence Vietnam's trade balance with respect to the UK in the long run, but USD/VND causes J-curve effect and thus supports the Marshall-Lerner condition. Moreover, robust support for the Marshall-Lerner condition is detected in the trade between Vietnam and Austria, Cyprus, France and Greece when VND depreciation against EUR or USD, regardless of the currency used, stimulates Vietnam's trade balance in the long run. On the contrary, we also find persuasive results for the unfavorable impacts of exchange rates, regardless of the currency used, on the trade balance of Vietnam with respect to some partners such as Belgium, Bulgaria, Germany, Hungary and Luxembourg.

The findings of this study can be useful for Vietnam's policy makers, especially when Vietnam was first regarded as currency manipulator by the USA in December 2020. Relating to this incident, the USA chided that Vietnam devaluated her currency against USD by more than 8% in recent years to encourage exportation, but Vietnam rejected that blame. In the process of solving this problem, cooperation between the two parties is very important and various measures can be made. For example, the adjustment of the foreign exchange management to let VND appreciate against USD may be required by the USA. If this happens, not only Vietnam-USA trade but also the trade between Vietnam and the EU-27 plus the UK as well as other partners such as China can be affected because USD is highly employed as vehicle currency. Our findings indicate that, when VND appreciates against USD, Vietnam's trade balance with 14 countries comprising more than 43.1% of the total Vietnam-EU trade value is facilitated. Meanwhile, Vietnam's trade balance with 7 countries accounting for only 8.1% of the total Vietnam-EU trade value is reduced. Consequently, the appreciation of VND against USD can be more beneficial to Vietnam's trade with the EU and the UK.

A considerable number of papers about exchange rate-trade balance relationship in general and J-curve effect in particular document no cointegration or insignificant results. Two culprits of this issue have been clearly indicated by the existing literature: aggregation bias, and symmetric assumption of exchange rate-trade balance linkage. In this paper, we suggest that the neglection of vehicle currency can be another culprit contributing to this problem of bilateral-level and commodity-level studies when examining the trade of a country, especially a developing one, with the partners that are different from the USA. And the empirical findings of our paper support this suggestion for the case of Vietnam when more significant short-run and long-run coefficients in USD models are found. Therefore, future studies using bilateral-level or commodity-level data can test this proposition for other countries by incorporating the role of USD as vehicle currency alongside the bilateral exchange rates. In addition, this approach seems very promising for research at commodity-level because USD has long been the world's leading vehicle currency and some commodities (such as crude oil) are usually invoiced in USD even if neither the exporters nor importers are the USA.

## Declarations

### Author contribution statement

Hoang Phong Le and Ho Hoang Gia Bao: Conceived and designed the experiments; Performed the experiments; Analyzed and interpreted the data; Contributed reagents, materials, analysis tools or data; Wrote the paper.

### Funding statement

This work was supported by the 10.13039/100010661European Union's Horizon 2020 research and innovation programme under 10.13039/100010665Marie Skłodowska-Curie grant agreement No. 734712. This work was also supported by the University of Economics Ho Chi Minh City.

### Data availability statement

Data will be made available on request.

### Declaration of interests statement

The authors declare no conflict of interest.

### Additional information

No additional information is available for this paper.

## Footnotes

1 Authors' calculation from the Direction of Trade Statistics data provided by IMF.

2 The problem of ignored vehicle currency normally does not happen in aggregate-level research because the USA is usually included in “the rest of the world” as she is a large trading partner of many countries. However, if the USA is not included, aggregate-level studies still face the weakness of omitting the role of USD as vehicle currency.

3 Due to the large number of variables used in this study, the unit root test results are not presented. The authors conducted the unit root tests and found no I(2) variable. Thus, the NARDL method is valid.
