# Supplementary material for: Asymmetric impact of exchange rate on trade between Vietnam and each of EU-27 countries and the UK: evidence from nonlinear ARDL and the role of vehicle currency
Source: Heliyon. 2021 Jun 19;7(6):e07344. doi: 10.1016/j.heliyon.2021.e07344 (PMC8239745; doi:10.1016/j.heliyon.2021.e07344)
Supplement: Appendix. Supplementary file [file mmc1.docx]

**Appendix.** Estimation results of bilateral exchange rate (BER) and vehicle currency exchange rate (USD) models

|  | i = Austria | | i = Belgium | | i = Bulgaria | |
| --- | --- | --- | --- | --- | --- | --- |
|  | BER | USD | BER | USD | BER | USD |
| **Long run** |  |  |  |  |  |  |
| POS | 8.47* | 44.67*** | -0.28 | -1.10 | -3.03** | -9.85*** |
| NEG | -0.53 | 21.76*** | -0.34** | -3.13** | 0.26 | -4.10* |
| GDP | -2.07 | -14.16*** | 1.60*** | 3.14*** | -1.94*** | 3.12* |
| GDPF | -32.78** | 24.39** | -0.70 | -7.42** | 4.61* | -2.93* |
| **Short run** |  |  |  |  |  |  |
| **ΔPOS_t_** | -0.28 | 10.74 | -0.39 | 1.83 | -6.06** | 17.86 |
| ΔPOS_t-1_ | -1.12 | -14.76** |  | -3.51 |  | 36.97** |
| ΔPOS_t-2_ | 0.32 | -19.73*** |  |  |  | 11.34 |
| ΔPOS_t-3_ | 3.91** | 8.75 |  |  |  | 15.45 |
| ΔPOS_t-4_ |  |  |  |  |  | 26.56** |
| ΔPOS_t-5_ |  |  |  |  |  | -33.87*** |
| ΔPOS_t-6_ |  |  |  |  |  | -16.05 |
| ΔPOS_t-7_ |  |  |  |  |  |  |
| **ΔNEG_t_** | -0.14 | -1.75 | -0.47** | -2.65** | 0.29 | -5.19 |
| ΔNEG_t-1_ |  | -5.20 |  |  | -1.20 | -16.71 |
| ΔNEG_t-2_ |  | 7.52 |  |  | 5.96** | -32.11*** |
| ΔNEG_t-3_ |  |  |  |  |  | -50.72*** |
| ΔNEG_t-4_ |  |  |  |  |  | -58.59*** |
| ΔNEG_t-5_ |  |  |  |  |  | -19.35 |
| ΔNEG_t-6_ |  |  |  |  |  |  |
| ΔNEG_t-7_ |  |  |  |  |  |  |
| **ΔGDP_t_** | -0.53 | -1.65*** | 0.21*** | 0.27 | -1.52*** | 3.60 |
| ΔGDP_t-1_ |  | 3.18*** | -1.15*** | -1.32*** |  | 3.56 |
| ΔGDP_t-2_ |  | 1.26** | -0.93 | -1.16*** |  | 19.08** |
| ΔGDP_t-3_ |  |  |  |  |  | 26.93*** |
| ΔGDP_t-4_ |  |  |  |  |  | 20.47** |
| ΔGDP_t-5_ |  |  |  |  |  | 13.45* |
| ΔGDP_t-6_ |  |  |  |  |  | 1.05 |
| ΔGDP_t-7_ |  |  |  |  |  | -4.54 |
| **ΔGDPF_t_** | -14.89 | -8.98 | -12.20** | -12.59** | 5.60 | 8.41 |
| ΔGDPF_t-1_ | -2.26 | -25.15* |  | 9.59* | -22.51** | -24.44 |
| ΔGDPF_t-2_ | 9.44 |  |  | -1.03 | 20.46** | 15.57 |
| ΔGDPF_t-3_ | 26.84** |  |  | 11.75** |  | 8.47 |
| ΔGDPF_t-4_ |  |  |  |  |  | 13.23 |
| ΔGDPF_t-5_ |  |  |  |  |  | -30.81** |
| ΔGDPF_t-6_ |  |  |  |  |  |  |
| ΔGDPF_t-7_ |  |  |  |  |  |  |
| Constant | 41.8** | -20.51 | -0.31 | 19.80** | -3.69 | 16.98 |
| Bound test | 4.11** | 6.79*** | 5.24*** | 5.11*** | 13.30*** | 12.78*** |
| Adj – R^2^ | 0.73 | 0.76 | 0.74 | 0.74 | 0.64 | 0.79 |
| Breusch-Godfrey | 1.88 | 0.31 | 0.18 | 0.07 | 1.98 | 0.02 |
| Breusch–Pagan | 0.85 | 1.12 | 1.18 | 0.93 | 1.26 | 0.60 |
| Ramsey RESET | 0.50 | 1.75 | 0.13 | 0.22 | 0.06 | 1.44 |
| CUSUM | S | S | S | S | S | S |
| CUSUMSQ | S | S | S | S | S | S |

|  | i = Croatia | | i = Cyprus | | i = Czechia | |
| --- | --- | --- | --- | --- | --- | --- |
|  | BER | USD | BER | USD | BER | USD |
| **Long run** |  |  |  |  |  |  |
| POS | 0.03 | 0.05 | 0.74* | 6.36*** | -1.06* | 1.75 |
| NEG | 0.11*** | 0.38*** | 0.66** | 2.81*** | -0.27 | 1.42 |
| GDP | -0.23*** | -0.52*** | -4.14*** | -2.92*** | -0.50 | -1.16 |
| GDPF | 0.35*** | 0.65*** | -0.31 | 0.17 | 1.45 | -0.43 |
| **Short run** |  |  |  |  |  |  |
| **ΔPOS_t_** | 0.15 | 0.31 | 1.00 | 9.94*** | 0.54 | 1.19 |
| ΔPOS_t-1_ |  |  | 4.88** |  | 2.67** |  |
| ΔPOS_t-2_ |  |  |  |  |  |  |
| ΔPOS_t-3_ |  |  |  |  |  |  |
| ΔPOS_t-4_ |  |  |  |  |  |  |
| ΔPOS_t-5_ |  |  |  |  |  |  |
| ΔPOS_t-6_ |  |  |  |  |  |  |
| ΔPOS_t-7_ |  |  |  |  |  |  |
| **ΔNEG_t_** | 0.003 | 0.23 | 0.88** | -7.91* | -1.80 | -0.65 |
| ΔNEG_t-1_ |  | -0.35* |  |  | -2.78* | -8.03** |
| ΔNEG_t-2_ |  | -0.05 |  |  |  |  |
| ΔNEG_t-3_ |  | 0.45** |  |  |  |  |
| ΔNEG_t-4_ |  |  |  |  |  |  |
| ΔNEG_t-5_ |  |  |  |  |  |  |
| ΔNEG_t-6_ |  |  |  |  |  |  |
| ΔNEG_t--7_ |  |  |  |  |  |  |
| **ΔGDP_t_** | -0.14*** | -0.12* | -1.93** | -1.61*** | -0.46 | -0.79 |
| ΔGDP_t-1_ | 0.08* | 0.20*** | 3.85*** | 3.29*** |  |  |
| ΔGDP_t-2_ | 0.10*** | 0.09* |  |  |  |  |
| ΔGDP_t-3_ |  |  |  |  |  |  |
| ΔGDP_t-4_ |  |  |  |  |  |  |
| ΔGDP_t-5_ |  |  |  |  |  |  |
| ΔGDP_t-6_ |  |  |  |  |  |  |
| ΔGDP_t-7_ |  |  |  |  |  |  |
| **ΔGDPF_t_** | 0.29*** | 0.21 | 11.56** | 4.10 | -1.45 | -0.29 |
| ΔGDPF_t-1_ |  | -0.30** | -6.05 | -10.57*** | -2.47** |  |
| ΔGDPF_t-2_ |  |  | 8.58 |  |  |  |
| ΔGDPF_t-3_ |  |  | 6.80 |  |  |  |
| ΔGDPF_t-4_ |  |  |  |  |  |  |
| ΔGDPF_t-5_ |  |  |  |  |  |  |
| ΔGDPF_t-6_ |  |  |  |  |  |  |
| ΔGDPF_t-7_ |  |  |  |  |  |  |
| Constant | -0.40** | -0.43** | 33.65*** | 25.89*** | -0.32 | 7.73* |
| Bound test | 4.53*** | 3.82** | 4.74*** | 6.68*** | 6.01*** | 6.05*** |
| Adj – R^2^ | 0.74 | 0.78 | 0.63 | 0.66 | 0.56 | 0.53 |
| Breusch-Godfrey | 1.35 | 0.78 | 0.63 | 0.79 | 0.21 | 0.55 |
| Breusch–Pagan | 1.29 | 0.80 | 1.59 | 1.08 | 0.94 | 0.37 |
| Ramsey RESET | 0.31 | 1.56 | 2.14 | 0.12 | 0.04 | 0.00 |
| CUSUM | S | S | S | S | S | S |
| CUSUMSQ | U | S | S | S | S | S |

|  | i = Denmark | | i = Estonia | | i = Finland | |
| --- | --- | --- | --- | --- | --- | --- |
|  | BER | USD | BER | USD | BER | USD |
| **Long run** |  |  |  |  |  |  |
| POS | 1.27*** | -0.34 | -0.000 | 0.06 | 0.19 | -0.41 |
| NEG | 0.75*** | -0.36 | -0.018** | -0.02 | 0.24 | 2.35* |
| GDP | -0.15 | -0.25 | 0.06** | 0.06** | 0.27 | -3.01** |
| GDPF | -5.87*** | -6.27*** | -0.04*** | -0.04** | 2.01 | 6.55*** |
| **Short run** |  |  |  |  |  |  |
| **ΔPOS_t_** | 1.26*** | 2.75 | -0.00 | 0.03 | 0.19 | -1.01 |
| ΔPOS_t-1_ |  | -15.15*** |  |  |  | -2.92 |
| ΔPOS_t-2_ |  |  |  |  |  | -10.77** |
| ΔPOS_t-3_ |  |  |  |  |  |  |
| ΔPOS_t-4_ |  |  |  |  |  |  |
| ΔPOS_t-5_ |  |  |  |  |  |  |
| ΔPOS_t-6_ |  |  |  |  |  |  |
| ΔPOS_t-7_ |  |  |  |  |  |  |
| **ΔNEG_t_** | -1.18 | -4.21* | -0.01* | -0.01 | -0.07 | 4.30 |
| ΔNEG_t-1_ |  |  |  |  | -2.81** | 10.47** |
| ΔNEG_t-2_ |  |  |  |  | -1.02 | 16.18*** |
| ΔNEG_t-3_ |  |  |  |  | 0.20 | 10.41** |
| ΔNEG_t-4_ |  |  |  |  | -3.36*** |  |
| ΔNEG_t-5_ |  |  |  |  |  |  |
| ΔNEG_t-6_ |  |  |  |  |  |  |
| ΔNEG_t-7_ |  |  |  |  |  |  |
| **ΔGDP_t_** | -0.15 | -0.17 | 0.01 | 0.03* | 0.27 | -5.52*** |
| ΔGDP_t-1_ |  |  | -0.02** |  |  | -2.48 |
| ΔGDP_t-2_ |  |  | -0.01** |  |  | -4.56** |
| ΔGDP_t-3_ |  |  |  |  |  | -4.64** |
| ΔGDP_t-4_ |  |  |  |  |  |  |
| ΔGDP_t-5_ |  |  |  |  |  |  |
| ΔGDP_t-6_ |  |  |  |  |  |  |
| ΔGDP_t-7_ |  |  |  |  |  |  |
| **ΔGDPF_t_** | -5.81*** | -4.11*** | -0.02** | -0.02* | -5.54*** | -3.05 |
| ΔGDPF_t-1_ |  |  |  |  |  | -5.44* |
| ΔGDPF_t-2_ |  |  |  |  |  |  |
| ΔGDPF_t-3_ |  |  |  |  |  |  |
| ΔGDPF_t-4_ |  |  |  |  |  |  |
| ΔGDPF_t-5_ |  |  |  |  |  |  |
| ΔGDPF_t-6_ |  |  |  |  |  |  |
| ΔGDPF_t-7_ |  |  |  |  |  |  |
| Constant | 30.68*** | 22.24*** | -0.05 | -0.05 | -7.52 | -13.24* |
| Bound test | 7.31*** | 4.83*** | 3.58** | 3.43* | 11.48*** | 12.24*** |
| Adj – R^2^ | 0.70 | 0.73 | 0.26 | 0.25 | 0.79 | 0.80 |
| Breusch-Godfrey | 1.40 | 0.14 | 0.39 | 0.97 | 0.64 | 0.25 |
| Breusch–Pagan | 2.23** | 0.69 | 0.75 | 0.98 | 0.65 | 1.09 |
| Ramsey RESET | 1.74 | 0.08 | 1.13 | 2.51 | 3.39* | 2.04 |
| CUSUM | U | S | S | S | S | S |
| CUSUMSQ | S | S | S | S | S | S |

|  | i = France | | i = Germany | | i = Greece | |
| --- | --- | --- | --- | --- | --- | --- |
|  | BER | USD | BER | USD | BER | USD |
| **Long run** |  |  |  |  |  |  |
| POS | 1.11*** | 4.12** | -1.11*** | -2.65* | 3.18*** | 12.98** |
| NEG | -0.39* | 5.22*** | -0.67** | 0.77 | 3.32*** | 8.51*** |
| GDP | -0.78** | -5.23*** | -0.50 | -1.71*** | 0.98 | -7.60*** |
| GDPF | -12.13*** | 11.25*** | 0.90 | 4.57** | -3.33*** | 4.43*** |
| **Short run** |  |  |  |  |  |  |
| **ΔPOS_t_** | -4.10*** | -4.84 | 0.09 | -1.73 | 3.47 | 8.12** |
| ΔPOS_t-1_ | -5.84*** | -8.15** | -0.93 | -7.47 |  |  |
| ΔPOS_t-2_ | -6.33*** | -5.83* | -1.88* | -12.14** |  |  |
| ΔPOS_t-3_ | -8.87*** | -15.29*** | -1.88* | 8.86* |  |  |
| ΔPOS_t-4_ | -6.74*** | -0.67 |  | 10.66* |  |  |
| ΔPOS_t-5_ | -6.44*** | -1.71 |  | -1.36 |  |  |
| ΔPOS_t-6_ | -4.22*** | -6.67** |  | -9.72* |  |  |
| ΔPOS_t-7_ | -2.37* |  |  |  |  |  |
| **ΔNEG_t_** | 4.11*** | -6.91*** | -0.61* | 14.02*** | 1.04 | 5.32*** |
| ΔNEG_t-1_ | 4.20*** |  |  | 20.16*** | -9.09*** |  |
| ΔNEG_t-2_ | 3.21** |  |  | 11.00 | -6.76 |  |
| ΔNEG_t-3_ | 4.22*** |  |  | -6.70 | -14.01*** |  |
| ΔNEG_t-4_ | 2.33** |  |  |  | -13.27*** |  |
| ΔNEG_t-5_ | 1.18 |  |  |  | -8.31** |  |
| ΔNEG_t-6_ | 1.20 |  |  |  | 0.37 |  |
| ΔNEG_t-7_ | 0.92 |  |  |  | -6.98** |  |
| **ΔGDP_t_** | 0.58 | 4.41*** | -0.79*** | -7.67** | -8.18** | -3.72*** |
| ΔGDP_t-1_ | -1.39 | 3.61*** |  | -1.87 | -7.97** |  |
| ΔGDP_t-2_ | -1.68 | 2.29** |  | -1.21 | -6.30* |  |
| ΔGDP_t-3_ | -1.73 | 1.25* |  | 5.03*** | -5.65* |  |
| ΔGDP_t-4_ | -3.74** |  |  | 11.18*** |  |  |
| ΔGDP_t-5_ |  |  |  | 7.95*** |  |  |
| ΔGDP_t-6_ |  |  |  | 6.73*** |  |  |
| ΔGDP_t-7_ |  |  |  |  |  |  |
| ΔGDP_t-8_ |  |  |  |  |  |  |
| **ΔGDPF_t_** | -5.11 | -18.23** | 0.82 | 20.03** | 3.28 | 2.77*** |
| ΔGDPF_t-1_ | 15.12 |  |  | -9.28 | 16.23*** |  |
| ΔGDPF_t-2_ | 30.41*** |  |  | -9.44 | 15.81*** |  |
| ΔGDPF_t-3_ | 29.99*** |  |  | -8.71 | 21.97*** |  |
| ΔGDPF_t-4_ | 7.50 |  |  | 4.12 | 16.24*** |  |
| ΔGDPF_t-5_ | 20.69** |  |  | 6.55 | 10.94** |  |
| ΔGDPF_t-6_ | 28.07*** |  |  | 10.95* |  |  |
| ΔGDPF_t-7_ | 15.15 |  |  |  |  |  |
| Constant | 134.21*** | -22.82** | 2.30 | -19.31 | -17.40 | 10.25** |
| Bound test | 7.89*** | 7.31*** | 4.73*** | 4.23** | 6.53*** | 9.39*** |
| Adj – R^2^ | 0.83 | 0.76 | 0.71 | 0.77 | 0.71 | 0.57 |
| Breusch-Godfrey | 0.20 | 0.95 | 0.13 | 1.23 | 2.21 | 0.91 |
| Breusch–Pagan | 0.77 | 1.03 | 0.88 | 1.44 | 1.41 | 1.21 |
| Ramsey RESET | 1.77 | 2.13 | 0.00 | 0.56 | 0.68 | 0.13 |
| CUSUM | S | S | S | S | S | S |
| CUSUMSQ | S | S | S | S | S | U |

|  | i = Hungary | | i = Ireland | | i = Italy | |
| --- | --- | --- | --- | --- | --- | --- |
|  | BER | USD | BER | USD | BER | USD |
| **Long run** |  |  |  |  |  |  |
| POS | -3.44 | -2.36 | -0.70 | -3.16* | 0.74 | 3.13 |
| NEG | -5.61** | -12.97*** | 0.33 | 4.19*** | 0.39 | 2.27 |
| GDP | 14.94** | 19.40*** | 1.62 | -4.17*** | -1.83* | -6.10*** |
| GDPF | -4.77 | -13.56** | 2.75* | 2.67*** | -0.12 | 6.37* |
| **Short run** |  |  |  |  |  |  |
| **ΔPOS_t_** | -0.62** | -3.03 | -0.05 | -4.57 | -1.82* | 4.29 |
| ΔPOS_t-1_ |  | -5.69 | 0.99 | -5.53 | -2.22** | -10.13** |
| ΔPOS_t-2_ |  | 14.67*** | 0.01 | -4.29 | -1.08 | -8.33* |
| ΔPOS_t-3_ |  | 9.02** | -0.75 | 6.41 | -0.23 | -7.63 |
| ΔPOS_t-4_ |  |  | 2.80** | 6.21 | 0.53 | 0.21 |
| ΔPOS_t-5_ |  |  | 2.65* |  | 1.71** | -4.32 |
| ΔPOS_t-6_ |  |  | 3.34** |  | 0.14 | -11.06** |
| ΔPOS_t-7_ |  |  |  |  | 1.89** | -6.36 |
| **ΔNEG_t_** | -2.39*** | 2.60 | 0.18 | -7.55** | 0.15 | 5.89 |
| ΔNEG_t-1_ |  | 6.42** |  | -12.79*** |  | 11.95** |
| ΔNEG_t-2_ |  |  |  | 5.53 |  | 4.83 |
| ΔNEG_t-3_ |  |  |  |  |  | -4.47 |
| ΔNEG_t-4_ |  |  |  |  |  | -23.99*** |
| ΔNEG_t-5_ |  |  |  |  |  | -23.49*** |
| ΔNEG_t-6_ |  |  |  |  |  | -11.43** |
| ΔNEG_t-7_ |  |  |  |  |  |  |
| **ΔGDP_t_** | 2.68*** | 2.55** | 0.26 | -1.80** | -0.69*** | -8.12*** |
| ΔGDP_t-1_ |  | -2.21*** |  | 5.58*** |  | -2.64 |
| ΔGDP_t-2_ |  | -1.58*** |  | 2.62*** |  | 1.19 |
| ΔGDP_t-3_ |  |  |  |  |  | 6.88 |
| ΔGDP_t-4_ |  |  |  |  |  | 12.80*** |
| ΔGDP_t-5_ |  |  |  |  |  | 13.33*** |
| ΔGDP_t-6_ |  |  |  |  |  | 7.37*** |
| ΔGDP_t-7_ |  |  |  |  |  |  |
| **ΔGDPF_t_** | -6.49*** | -4.56** | 1.63 | 2.51** | 7.06* | 10.12 |
| ΔGDPF_t-1_ | -2.15** |  | 2.43* | -0.79 |  | 12.18 |
| ΔGDPF_t-2_ | -2.87*** |  | 1.03 | -1.04 |  | 3.37 |
| ΔGDPF_t-3_ |  |  | -0.19 | -1.41 |  | 7.60 |
| ΔGDPF_t-4_ |  |  | -6.03*** | -6.66*** |  | -1.29 |
| ΔGDPF_t-5_ |  |  | -4.88*** | -5.79*** |  | 20.67** |
| ΔGDPF_t-6_ |  |  | -3.69** | -5.95*** |  |  |
| ΔGDPF_t-7_ |  |  |  | -1.86 |  |  |
| Constant | -8.15** | -8.98** | -9.55*** | 26.30** | 5.10 | 5.03 |
| Bound test | 4.98*** | 3.83** | 5.26*** | 4.59*** | 3.79** | 5.2*** |
| Adj – R^2^ | 0.60 | 0.61 | 0.69 | 0.71 | 0.88 | 0.89 |
| Breusch-Godfrey | 0.24 | 0.37 | 1.49 | 1.17 | 0.78 | 3.10 |
| Breusch–Pagan | 1.40 | 1.24 | 1.37 | 0.91 | 1.03 | 0.53 |
| Ramsey RESET | 0.69 | 1.77 | 1.78 | 1.44 | 4.48** | 0.80 |
| CUSUM | S | S | S | S | S | S |
| CUSUMSQ | S | S | S | S | U | S |

|  | i = Latvia | | i = Lithuania | | i = Luxembourg | |
| --- | --- | --- | --- | --- | --- | --- |
|  | BER | USD | BER | USD | BER | USD |
| **Long run** |  |  |  |  |  |  |
| POS | 5.25 | 89.29*** | 0.55 | -32.50*** | -5.50** | -4.73 |
| NEG | 0.32 | -16.96** | 4.73*** | -10.86** | -7.70*** | -42.47** |
| GDP | -4.05 | 3.84 | -4.54** | 5.58 | -1.61 | 28.50 |
| GDPF | -9.17 | -4.63 | 3.49** | -1.36 | -10.71 | -48.67** |
| **Short run** |  |  |  |  |  |  |
| **ΔPOS_t_** | 0.79 | 2.61 | 0.44 | -26.41*** | -3.44** | 26.71 |
| ΔPOS_t-1_ |  | -26.07** |  |  |  | 6.87 |
| ΔPOS_t-2_ |  | -15.13 |  |  |  | 44.07** |
| ΔPOS_t-3_ |  | -20.91** |  |  |  |  |
| ΔPOS_t-4_ |  |  |  |  |  |  |
| ΔPOS_t-5_ |  |  |  |  |  |  |
| ΔPOS_t-6_ |  |  |  |  |  |  |
| ΔPOS_t-7_ |  |  |  |  |  |  |
| **ΔNEG_t_** | 0.05 | -5.57** | 3.78*** | -8.82** | -4.81*** | -16.88 |
| ΔNEG_t-1_ |  |  |  |  |  | -26.86 |
| ΔNEG_t-2_ |  |  |  |  |  | -41.61** |
| ΔNEG_t-3_ |  |  |  |  |  |  |
| ΔNEG_t-4_ |  |  |  |  |  |  |
| ΔNEG_t-5_ |  |  |  |  |  |  |
| ΔNEG_t-6_ |  |  |  |  |  |  |
| ΔNEG_t-7_ |  |  |  |  |  |  |
| **ΔGDP_t_** | -0.61 | 1.56 | -1.48* | 1.82 | -1.01 | 18.51** |
| ΔGDP_t-1_ |  |  | 1.86** |  |  | 8.04 |
| ΔGDP_t-2_ |  |  |  |  |  | 11.41* |
| ΔGDP_t-3_ |  |  |  |  |  | 16.42** |
| ΔGDP_t-4_ |  |  |  |  |  |  |
| ΔGDP_t-5_ |  |  |  |  |  |  |
| ΔGDP_t-6_ |  |  |  |  |  |  |
| ΔGDP_t-7_ |  |  |  |  |  |  |
| **ΔGDPF_t_** | 0.95 | -1.52 | 2.79*** | 4.19 | -6.69 | -9.65 |
| ΔGDPF_t-1_ | 1.82 |  |  | 6.04** |  |  |
| ΔGDPF_t-2_ | 1.91* |  |  | -0.03 |  |  |
| ΔGDPF_t-3_ |  |  |  | 7.32** |  |  |
| ΔGDPF_t-4_ |  |  |  |  |  |  |
| ΔGDPF_t-5_ |  |  |  |  |  |  |
| ΔGDPF_t-6_ |  |  |  |  |  |  |
| ΔGDPF_t-7_ |  |  |  |  |  |  |
| Constant | 10.12* | 0.96 | 7.13 | -10.83 | 37.07** | 43.68** |
| Bound test | 1.6 | 4.22* | 9.18*** | 10.14*** | 7.00*** | 4.89*** |
| Adj – R^2^ | 0.12 | 0.21 | 0.48 | 0.57 | 0.39 | 0.47 |
| Breusch-Godfrey | 0.16 | 0.22 | 0.87 | 0.90 | 1.18 | 1.32 |
| Breusch–Pagan | 1.35 | 1.04 | 0.90 | 0.77 | 1.57 | 1.10 |
| Ramsey RESET | 2.95* | 1.74 | 7.56*** | 2.75 | 0.40 | 0.27 |
| CUSUM | S | S | U | S | S | S |
| CUSUMSQ | U | S | S | S | U | U |

|  | i = Malta | | | | i = Netherlands | | | | | i = Poland | | |
| --- | --- | --- | --- | --- | --- | --- | --- | --- | --- | --- | --- | --- |
|  | BER | | USD | | BER | | | USD | | BER | | USD |
| **Long run** |  | |  | |  | | |  | |  | |  |
| POS | 0.01 | | -0.16 | | -1.24 | | | 8.48 | | -2.10*** | | 10.43** |
| NEG | -0.003 | | 0.22* | | -2.05** | | | 4.77 | | -0.33 | | 0.30 |
| GDP | 0.03** | | -0.07 | | -1.41 | | | -9.89 | | -1.38** | | -0.11 |
| GDPF | -0.03* | | -0.08*** | | -7.67 | | | 1.02 | | 2.88* | | 5.05** |
| **Short run** |  | |  | |  | | |  | |  | |  |
| **ΔPOS_t_** | 0.02 | | 0.27 | | -0.21 | | | 2.02 | | -1.72*** | | 7.43** |
| ΔPOS_t-1_ | 0.06** | | 0.62*** | |  | | | -3.48* | |  | |  |
| ΔPOS_t-2_ | 0.03 | | 0.66*** | |  | | | -2.66 | |  | |  |
| ΔPOS_t-3_ | -0.04 | | 0.02 | |  | | |  | |  | |  |
| ΔPOS_t-4_ |  | | 0.47** | |  | | |  | |  | |  |
| ΔPOS_t-5_ |  | | 0.10 | |  | | |  | |  | |  |
| ΔPOS_t-6_ |  | | 0.37** | |  | | |  | |  | |  |
| ΔPOS_t-7_ |  | | 0.21 | |  | | |  | |  | |  |
| **ΔNEG_t_** | -0.003 | | 0.21* | | -0.35* | | | 0.49 | | -0.27 | | 0.21 |
| ΔNEG_t-1_ |  | | -0.58** | |  | | |  | |  | |  |
| ΔNEG_t-2_ |  | | -0.69** | |  | | |  | |  | |  |
| ΔNEG_t-3_ |  | | -0.07 | |  | | |  | |  | |  |
| ΔNEG_t-4_ |  | | -0.04 | |  | | |  | |  | |  |
| ΔNEG_t-5_ |  | | -0.14 | |  | | |  | |  | |  |
| ΔNEG_t-6_ |  | | -0.32 | |  | | |  | |  | |  |
| ΔNEG_t-7_ |  | |  | |  | | |  | |  | |  |
| **ΔGDP_t_** | 0.01 | | 0.22** | | -0.24* | | | -0.43** | | -1.13** | | -0.07 |
| ΔGDP_t-1_ |  | | 0.17 | |  | | | 0.41** | |  | |  |
| ΔGDP_t-2_ |  | | 0.19 | |  | | |  | |  | |  |
| ΔGDP_t-3_ |  | | 0.10 | |  | | |  | |  | |  |
| ΔGDP_t-4_ |  | | -0.01 | |  | | |  | |  | |  |
| ΔGDP_t-5_ |  | | 0.15 | |  | | |  | |  | |  |
| ΔGDP_t-6_ |  | | 0.06 | |  | | |  | |  | |  |
| ΔGDP_t-7_ |  | | 0.13** | |  | | |  | |  | |  |
| **ΔGDPF_t_** | 0.01 | | -0.13 | | 3.96 | | | 4.85 | | 2.36* | | 1.78 |
| ΔGDPF_t-1_ |  | | -0.06 | |  | | |  | |  | |  |
| ΔGDPF_t-2_ |  | | 0.10 | |  | | |  | |  | |  |
| ΔGDPF_t-3_ |  | | -0.02 | |  | | |  | |  | |  |
| ΔGDPF_t-4_ |  | | 0.01 | |  | | |  | |  | |  |
| ΔGDPF_t-5_ |  | | 0.17** | |  | | |  | |  | |  |
| ΔGDPF_t-6_ |  | | -0.07 | |  | | |  | |  | |  |
| ΔGDPF_t-7_ |  | | 0.10 | |  | | |  | |  | |  |
| Constant | -0.037 | | 1.39** | | 7.84* | | | 4.72 | | -2.72 | | -14.92* |
| Bound test | 9.52*** | | 7.56*** | | 4.64*** | | | 4.60*** | | 7.48*** | | 9.01*** |
| Adj – R^2^ | 0.54 | | 0.68 | | 0.62 | | | 0.59 | | 0.40 | | 0.40 |
| Breusch-Godfrey | 0.79 | | 0.50 | | 0.47 | | | 0.46 | | 1.09 | | 0.81 |
| Breusch–Pagan | 0.87 | | 0.66 | | 0.93 | | | 0.68 | | 2.51** | | 3.13*** |
| Ramsey RESET | 15.19*** | | 20.02*** | | 0.21 | | | 9.19*** | | 0.08 | | 1.62 |
| CUSUM | S | | S | | S | | | S | | S | | S |
| CUSUMSQ | U | | S | | S | | | S | | U | | U |
|  | | i = Portugal | | | | i = Romania | | | i = Slovakia | | | |
|  | | BER | | USD | | BER | USD | | BER | | USD | |
| **Long run** | |  | |  | |  |  | |  | |  | |
| POS | | 1.12*** | | -3.61* | | 8.48*** | 17.15 | | 0.61** | | 4.76 | |
| NEG | | 0.80 | | -3.32*** | | -5.56** | 40.75*** | | -9.71*** | | 7.44** | |
| GDP | | -5.89*** | | -0.43 | | 8.31** | -36.03*** | | 2.82 | | -5.24** | |
| GDPF | | -3.33 | | -11.29*** | | 9.31* | 32.14*** | | 20.34*** | | 6.32** | |
| **Short run** | |  | |  | |  |  | |  | |  | |
| **ΔPOS_t_** | | -2.26 | | -3.96* | | -4.00 | 26.15 | | 0.32*** | | 2.40 | |
| ΔPOS_t-1_ | |  | |  | | -5.08 | 16.44 | |  | |  | |
| ΔPOS_t-2_ | |  | |  | | 0.16 | 40.96** | |  | |  | |
| ΔPOS_t-3_ | |  | |  | | -8.85* | 27.62 | |  | |  | |
| ΔPOS_t-4_ | |  | |  | |  | 34.53* | |  | |  | |
| ΔPOS_t-5_ | |  | |  | |  |  | |  | |  | |
| ΔPOS_t-6_ | |  | |  | |  |  | |  | |  | |
| ΔPOS_t-7_ | |  | |  | |  |  | |  | |  | |
| **ΔNEG_t_** | | 6.65*** | | -3.64*** | | -3.50 | 31.85*** | | 0.901 | | -10.21** | |
| ΔNEG_t-1_ | | -0.82 | |  | | 6.07* | -1.71 | | 5.88*** | |  | |
| ΔNEG_t-2_ | | 1.09 | |  | |  | 1.23 | | 2.49 | |  | |
| ΔNEG_t-3_ | | 2.50* | |  | |  | -0.17 | | 3.90** | |  | |
| ΔNEG_t-4_ | | 3.57** | |  | |  | -35.42* | |  | |  | |
| ΔNEG_t-5_ | |  | |  | |  | -18.12 | |  | |  | |
| ΔNEG_t-6_ | |  | |  | |  |  | |  | |  | |
| ΔNEG_t-7_ | |  | |  | |  |  | |  | |  | |
| **ΔGDP_t_** | | -1.30 | | -0.47 | | -1.74 | -27.18** | | -0.96 | | 1.07 | |
| ΔGDP_t-1_ | | 4.43*** | |  | |  | 20.39 | |  | | 1.35*** | |
| ΔGDP_t-2_ | | 2.15** | |  | |  | 24.45** | |  | |  | |
| ΔGDP_t-3_ | |  | |  | |  | 9.14 | |  | |  | |
| ΔGDP_t-4_ | |  | |  | |  | 17.70* | |  | |  | |
| ΔGDP_t-5_ | |  | |  | |  | 10.44 | |  | |  | |
| ΔGDP_t-6_ | |  | |  | |  |  | |  | |  | |
| ΔGDP_t-7_ | |  | |  | |  |  | |  | |  | |
| **ΔGDPF_t_** | | 4.55 | | -0.27 | | -14.32** | 19.54** | | 7.91** | | 3.19*** | |
| ΔGDPF_t-1_ | | 6.34 | | 8.20*** | | -1.58 | -18.00** | | -3.83* | |  | |
| ΔGDPF_t-2_ | | 0.21 | |  | | -8.79** | -7.23 | | -3.77 | |  | |
| ΔGDPF_t-3_ | | 12.77** | |  | | -13.53*** |  | |  | |  | |
| ΔGDPF_t-4_ | |  | |  | | 12.98** |  | |  | |  | |
| ΔGDPF_t-5_ | |  | |  | | -17.88*** |  | |  | |  | |
| ΔGDPF_t-6_ | |  | |  | |  |  | |  | |  | |
| ΔGDPF_t-7_ | |  | |  | |  |  | |  | |  | |
| Constant | | 62.31*** | | 64.90*** | | -71.44** | 25.96 | | -55.22*** | | -1.10 | |
| Bound test | | 6.26*** | | 16.23*** | | 5.73*** | 3.37* | | 7.05*** | | 8.50*** | |
| Adj – R^2^ | | 0.80 | | 0.70 | | 0.61 | 0.57 | | 0.54 | | 0.47 | |
| Breusch-Godfrey | | 0.08 | | 0.22 | | 0.02 | 0.18 | | 0.33 | | 1.07 | |
| Breusch–Pagan | | 0.12 | | 1.38 | | 0.99 | 2.52*** | | 1.52 | | 1.24 | |
| Ramsey RESET | | 1.28 | | 0.09 | | 0.20 | 2.42 | | 0.08 | | 0.64 | |
| CUSUM | | S | | S | | U | S | | S | | S | |
| CUSUMSQ | | S | | U | | S | S | | S | | U | |

|  | i = Slovenia | | i = Spain | | i = Sweden | |
| --- | --- | --- | --- | --- | --- | --- |
|  | BER | USD | BER | USD | BER | USD |
| **Long run** |  |  |  |  |  |  |
| POS | -0.001 | 0.03 | 1.12** | -2.03 | 6.72* | 0.13 |
| NEG | 0.018*** | 0.06*** | 0.89*** | 2.90** | -5.41 | 14.46** |
| GDP | -0.02* | -0.03*** | -0.59 | -6.73*** | -1.60 | -12.42*** |
| GDPF | 0.05* | 0.05** | 0.95 | 8.43*** | -4.76 | 2.90 |
| **Short run** |  |  |  |  |  |  |
| **ΔPOS_t_** | -0.001 | 0.04 | -2.79*** | -0.34 | -2.55* | -8.62* |
| ΔPOS_t-1_ |  |  | -0.95 | -2.85 |  | -11.19** |
| ΔPOS_t-2_ |  |  | -2.02*** | -6.14* |  |  |
| ΔPOS_t-3_ |  |  | -1.28* | -10.56*** |  |  |
| ΔPOS_t-4_ |  |  |  |  |  |  |
| ΔPOS_t-5_ |  |  |  |  |  |  |
| ΔPOS_t-6_ |  |  |  |  |  |  |
| ΔPOS_t-7_ |  |  |  |  |  |  |
| **ΔNEG_t_** | 0.02*** | 0.08*** | 0.52*** | -4.34** | 2.55 | -7.26* |
| ΔNEG_t-1_ |  |  |  |  | 1.21 | -15.41** |
| ΔNEG_t-2_ |  |  |  |  | 0.52 | -15.39*** |
| ΔNEG_t-3_ |  |  |  |  | 2.69** |  |
| ΔNEG_t-4_ |  |  |  |  |  |  |
| ΔNEG_t-5_ |  |  |  |  |  |  |
| ΔNEG_t-6_ |  |  |  |  |  |  |
| ΔNEG_t-7_ |  |  |  |  |  |  |
| **ΔGDP_t_** | -0.02** | -0.03*** | 0.43** | 5.11** | -0.55* | 4.14** |
| ΔGDP_t-1_ |  |  |  | 3.41*** |  | 12.79*** |
| ΔGDP_t-2_ |  |  |  | 1..47* |  | 10.14*** |
| ΔGDP_t-3_ |  |  |  |  |  | 7.79*** |
| ΔGDP_t-4_ |  |  |  |  |  |  |
| ΔGDP_t-5_ |  |  |  |  |  |  |
| ΔGDP_t-6_ |  |  |  |  |  |  |
| ΔGDP_t-7_ |  |  |  |  |  |  |
| **ΔGDPF_t_** | 0.05* | 0.06** | -4.21*** | -2.19 | 4.12 | -7.43 |
| ΔGDPF_t-1_ |  |  |  | 12.74*** | 2.50 |  |
| ΔGDPF_t-2_ |  |  |  | 6.75** | -13.66** |  |
| ΔGDPF_t-3_ |  |  |  | 6.94** | -3.01 |  |
| ΔGDPF_t-4_ |  |  |  | -2.29 | 11.58** |  |
| ΔGDPF_t-5_ |  |  |  | -11.52*** |  |  |
| ΔGDPF_t-6_ |  |  |  |  |  |  |
| ΔGDPF_t-7_ |  |  |  |  |  |  |
| Constant | -0.11 | -0.10 | 1.83 | -1.98 | -3.25 | 48.06** |
| Bound test | 6.00*** | 5.84*** | 6.07*** | 5.59*** | 6.79*** | 10.96*** |
| Adj – R^2^ | 0.64 | 0.68 | 0.84 | 0.82 | 0.69 | 0.75 |
| Breusch-Godfrey | 0.68 | 1.19 | 1.74 | 0.29 | 0.43 | 1.69 |
| Breusch–Pagan | 6.65*** | 3.69*** | 0.90 | 0.98 | 1.19 | 0.62 |
| Ramsey RESET | 0.04 | 0.02 | 0.26 | 0.40 | 0.86 | 1.34 |
| CUSUM | S | S | S | S | S | S |
| CUSUMSQ | U | U | S | S | S | S |

|  | i = UK | |  |  |  |  |
| --- | --- | --- | --- | --- | --- | --- |
|  | BER | USD |  |  |  |  |
| **Long run** |  |  |  |  |  |  |
| POS | 3.55 | 6.96*** |  |  |  |  |
| NEG | 0.60 | 6.57*** |  |  |  |  |
| GDP | 2.06 | -5.44*** |  |  |  |  |
| GDPF | -9.79 | 9.30*** |  |  |  |  |
| **Short run** |  |  |  |  |  |  |
| **ΔPOS_t_** | -2.06* | -0.65 |  |  |  |  |
| ΔPOS_t-1_ | -2.70* | -7.35** |  |  |  |  |
| ΔPOS_t-2_ | -3.19** | -5.57* |  |  |  |  |
| ΔPOS_t-3_ | -2.73** | -2.79 |  |  |  |  |
| ΔPOS_t-4_ | -4.28*** | -5.37** |  |  |  |  |
| ΔPOS_t-5_ |  |  |  |  |  |  |
| ΔPOS_t-6_ |  |  |  |  |  |  |
| ΔPOS_t-7_ |  |  |  |  |  |  |
| **ΔNEG_t_** | 0.30 | -1.13 |  |  |  |  |
| ΔNEG_t-1_ |  |  |  |  |  |  |
| ΔNEG_t-2_ |  |  |  |  |  |  |
| ΔNEG_t-3_ |  |  |  |  |  |  |
| ΔNEG_t-4_ |  |  |  |  |  |  |
| ΔNEG_t-5_ |  |  |  |  |  |  |
| ΔNEG_t-6_ |  |  |  |  |  |  |
| ΔNEG_t-7_ |  |  |  |  |  |  |
| **ΔGDP_t_** | 2.58*** | 0.89 |  |  |  |  |
| ΔGDP_t-1_ | 1.73* | 4.67*** |  |  |  |  |
| ΔGDP_t-2_ | 2.14** | 3.42*** |  |  |  |  |
| ΔGDP_t-3_ | 2.56*** | 2.39*** |  |  |  |  |
| ΔGDP_t-4_ |  |  |  |  |  |  |
| ΔGDP_t-5_ |  |  |  |  |  |  |
| ΔGDP_t-6_ |  |  |  |  |  |  |
| ΔGDP_t-7_ |  |  |  |  |  |  |
| **ΔGDPF_t_** | 2.60 | -2.19 |  |  |  |  |
| ΔGDPF_t-1_ | -15.92* | -21.50*** |  |  |  |  |
| ΔGDPF_t-2_ | 1.92 |  |  |  |  |  |
| ΔGDPF_t-3_ | 3.81 |  |  |  |  |  |
| ΔGDPF_t-4_ | 16.29** |  |  |  |  |  |
| ΔGDPF_t-5_ |  |  |  |  |  |  |
| ΔGDPF_t-6_ |  |  |  |  |  |  |
| ΔGDPF_t-7_ |  |  |  |  |  |  |
| Constant | 20.10* | -11.56* |  |  |  |  |
| Bound test | 4.32** | 12.57*** |  |  |  |  |
| Adj – R^2^ | 0.55 | 0.59 |  |  |  |  |
| Breusch-Godfrey | 0.43 | 0.88 |  |  |  |  |
| Breusch–Pagan | 0.78 | 0.87 |  |  |  |  |
| Ramsey RESET | 0.03 | 0.58 |  |  |  |  |
| CUSUM | S | S |  |  |  |  |
| CUSUMSQ | S | S |  |  |  |  |

Notes: *, ** and *** respectively denote 10%, 5% and 1% significance level. The values of Bound test, Breusch-Godfrey, Breusch-Pagan and Ramsey RESET tests are F statistics. “S” and “U” respectively indicate “Stable” and “Unstable” results of CUSUM and CUSUMSQ tests.
